# Validation and Optimization of an Image-Based Screening Method Applied to the Study of Neuronal Processes on Nanogrooves

**DOI:** 10.3389/fncel.2018.00415

**Published:** 2018-11-06

**Authors:** Alex J. Bastiaens, Sijia Xie, Dana A. M. Mustafa, Jean-Philippe Frimat, Jaap M. J. den Toonder, Regina Luttge

**Affiliations:** ^1^Department of Mechanical Engineering, Microsystems Group and Institute for Complex Molecular Systems, Eindhoven University of Technology, Eindhoven, Netherlands; ^2^MESA+ Institute for Nanotechnology, University of Twente, Enschede, Netherlands

**Keywords:** SH-SY5Y cells, nanogrooves, neuronal differentiation, neuronal development, neurite development, high-content screening

## Abstract

Research on neuronal differentiation and neuronal network guidance induced through nanotopographical cues generates large datasets, and therefore the analysis of such data can be aided by automatable, unbiased image screening tools. To link such tools, we present an image-based screening method to evaluate the influence of nanogroove pattern dimensions on neuronal differentiation. This new method consists of combining neuronal feature detection software, here HCA-Vision, and a Frangi vesselness algorithm to calculate neurite alignment values and quantify morphological aspects of neurons, which are measured via neurite length, neuronal polarity, and neurite branching, for differentiated SH-SY5Y cells cultured on nanogrooved polydimethylsiloxane (PDMS) patterns in the 200–2000 nm range. The applicability of this method is confirmed by our results, which find that the level of alignment is dependent on nanogroove dimensions. Furthermore, the screening method reveals that differentiation and alignment are correlated. In particular, patterns with groove widths >200 nm and with a low ridge width to pattern period ratio have a quantifiable influence on alignment, neurite length, and polarity. In summary, the novel combination of software that forms a base for this statistical analysis method demonstrates good potential for evaluating tissue microarchitecture, which depends on subtle design variation in substrate topography. Using the screening method, we obtained automated and sensitive quantified readouts from large datasets.

## Introduction

In recent years, the use of nanotopographical cues has been studied to influence neuronal differentiation, including the guiding of neuronal outgrowths, which are referred to as neurites or neuronal processes. Neuronal differentiation is dependent on biochemical and topographical cues from the extracellular microenvironment, including specifically the ECM. Mimicking specific ECM properties can direct these neuronal processes and stimulate neuritogenesis and may result in unique tissue architectures. Since physical cues play a role in linking cells and guiding neurites in the ECM (Tessier-Lavigne and Goodman, 1996), such design strategies can be applied to implant surfaces and *in vitro* cell studies. To date, a range of different nanostructures has been demonstrated to alter the physical environment and influence neuronal differentiation, polarity, migration, and neurite orientation. Reviews by Hoffman-Kim et al. (2010) and Nguyen et al. (2016) can be referred to for an overview of findings on the use of varying materials, cell types, and structures at the micro- and nanoscale. Specifically, nanogrooves have been shown to influence neurite length and/or alignment (Rajnicek et al., 1997; Johansson et al., 2006; Bremus-Koebberling et al., 2012; Xie and Luttge, 2014), increase the percentage of multipolar cells with increasing ridge widths (Ferrari et al., 2011), and promote differentiation toward the neuronal lineage in the case of stem cells (Yim et al., 2007; Song et al., 2016).

We have shown previously that this knowledge can be applied to mechanically actuated *in vitro* brain models (Xie, 2016), while others have studied nanogrooves for nerve regeneration (Ferrari et al., 2011) and electronic interfacing with neurons (Johansson et al., 2006). For *in vitro* brain models, control over the alignment of neuronal cells through nanotopography can aid in creating directed neuronal network architecture, which is essential in mimicking the naturally occurring hierarchical and layered structures of the brain’s architecture reproducibly with high experimental yield. Nanotopography can therefore be exploited in the construction of *in vitro* brain models to direct signal transduction pathways in the neuronal network and create a simplified version of interconnected regions of the brain with different types of cells.

In order to apply such an advanced design strategy for *in vitro* culture of neuronal networks, it is necessary to analyze the architecture and extent of differentiation of neuronal cells on these nanostructures. As mentioned above, previous research has embraced quantitative parameters such as the percentage of differentiated cells, neurite length, cell polarity, degree of neurite branching, and neurite alignment, which indicate how nanostructures can influence neuronal processes. The availability of automated image analysis provides an in-depth set of information on the interdependency of these parameters; allows time-efficient data handling; and guarantees a robust, standardized analysis methodology amongst multiple experimenters.

In the present study, we validate and optimize such an automated image-based screening analysis method that can be applied to quantify the response of differentiation and neurite alignment of SH-SY5Y cells on different nanogrooved patterns; such a method has not been performed before. The advantage of using the developed method is the ability to perform time efficient, unbiased, and automatable image analysis on a large dataset. Previously, studies used whole image FFT to determine the alignment of neuronal outgrowths in neuronal cell cultures (Johansson et al., 2006; Tonazzini et al., 2014; Xie, 2016). Here, the Frangi vesselness algorithm is applied to neurite-only images and introduced as a new method to quantify the degree of alignment. We validated this new method against neurite-only FFT and manual alignment measurements for SH-SY5Y cell cultures on nanogrooved patterns, using flat samples as a control. The vesselness algorithm yielded a linear correlation with a higher sensitivity than FFT. Therefore, we selected the vesselness algorithm to be combined with automated image analysis software specialized toward images of neuronal cells, HCA-Vision, in the method development process. We were particularly interested in quantitative observations of neurite length, neuronal polarity, neurite branching, and the correlation of these output variables, which together with the neurite alignment detail the neuronal response to the generated nanotopographies. Applying our new, automated method confirms with the other studies mentioned previously, that the influence of nanogrooved patterns on neurite alignment and neuronal differentiation properties differs compared to flat controls and in between patterns. The method was able to find these quantitative results with such a sensitivity that subtle differences between nanogrooved patterns in the range of 200–2000 nm can be observed.

## Materials and Methods

### Fabrication and Preparation of Nanogrooved PDMS Substrates

Substrates with nanogroove features were fabricated by replication from a cyclic olefin copolymer (COC; Kunststoff-Zentrum, Leipzig, Germany) template. Resist scaffolds were realized by J-FIL on silicon substrates as described by Xie and Luttge (2014). Nanogrooved pattern periodicity ranged from 200 to 2000 nm and ridge width was between 100 and 1340 nm in different combinations to produce 27 different patterns in total. Subsequently, a negative imprint master of COC was made by thermal nanoimprinting at 108°C under a pressure of 4 MPa on the resist scaffold. After cooling the COC template to room temperature it was peeled off the resist scaffold. The final nanogrooved substrates were made by so called soft lithography of polydimethylsiloxane (PDMS; Sylgard 184, Dow Corning, Wiesbaden, Germany). PDMS elastomer and curing agent were mixed in a 10:1 weight ratio and degassed for 10 min using a vacuum chamber prior to spin coating a 100 μm layer of PDMS onto the COC template and placing the PDMS-covered template into an oven at 65°C for 4 h to fully cure the PDMS.

**FIGURE 1:**
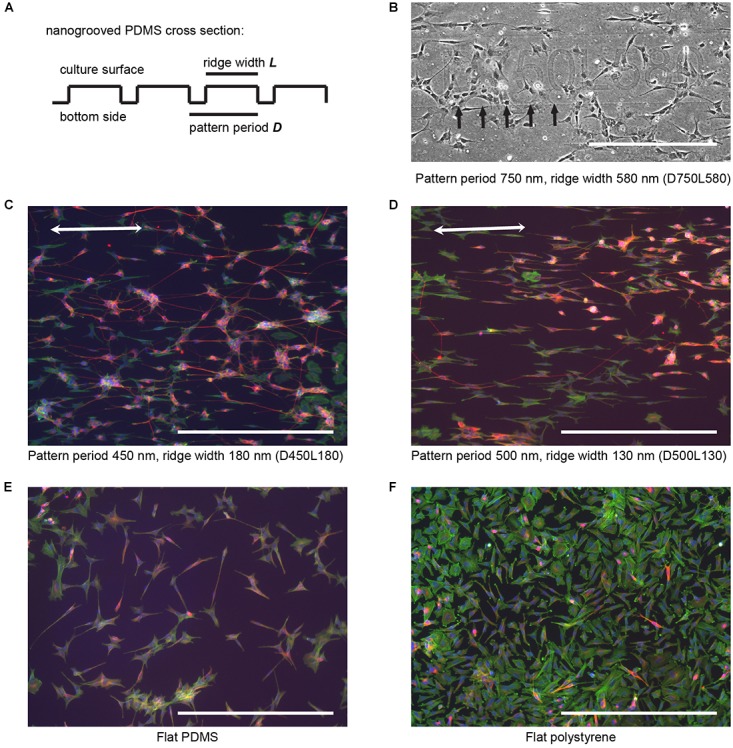
SH-SY5Y cell culture and differentiation on patterned and flat substrates. **(A)** Simplified cross section view for general fabricated nanogrooved patterns in PDMS. Nanogrooves in PDMS consist of a periodically repeating groove and ridge on the culture surface. For distinction between the nanogrooved patterns, a notation was used, where D and the subsequent value refer to pattern period and the respective size in nm and L and the subsequent value refer to the pattern ridge width and the respective size in nm, as is shown in **(B–F)**. **(B)** Section of an example image from pattern D750L580 of how samples for each nanogrooved pattern were identified. For each nanogrooved pattern in each experiment, an image of the pattern name as described in **(A)** and edge of the pattern were taken. Nanogroove direction is parallel to the edge seen underneath the pattern notation and should ideally be along the horizontal axis; however, due to the manual placement of samples underneath the microscope, slight deviations occur. Therefore, the images with pattern name and border were used as references to determine the exact orientation of the nanogrooves for each individual pattern by calculating the border angle in Fiji software. Brightness and contrast were enhanced for clarification of this example only; no editing was used for data gathering. Arrows denote pattern edge, and the scale bar represents 400 μm. **(C,D)** SH-SY5Y cells were cultured on top of PDMS with a pattern of 450 nm period and 180 nm ridge width **(C)** and 500 nm period and 130 nm ridge width **(D)**, and then they were differentiated using 10 μM RA and 50 ng ml^-1^ BDNF. Cells were stained for F-actin (green), β-Tubulin III (red), and cell nuclei (blue). Double-sided arrows denote the pattern direction, and the scale bar represents 400 μm. **(E,F)** SH-SY5Y cells were cultured on top of a flat PDMS substrate **(E)** and flat polystyrene substrate **(F)**, and then they were differentiated using 10 μM RA and 50 ng ml^-1^ BDNF. Cells were stained for F-actin (green), β-Tubulin III (red), and cell nuclei (blue). The scale bar represents 400 μm.

Nanogrooved PDMS (Figure 1A) was peeled off the template. The PDMS substrate includes a flat piece of PDMS of roughly 1 cm^2^ as a control surface. PDMS substrates were placed in polystyrene Petri dishes and sterilized for 5 min using 70% ethanol (VWR, Amsterdam, Netherlands), after which the substrates were placed in an oven at 65°C for 1 h to ensure that the ethanol evaporated. A fibronectin coating was applied by dispensing a solution of 20 μg ml^-1^ (Sigma-Aldrich, Zwijndrecht, Netherlands) diluted in phosphate buffered saline (PBS; Westburg, Leusden, Netherlands) onto the PDMS nanogrooves, flat surface, and Petri dish surface prior to seeding cells in the Petri dish for 30 min. Petri dishes with PDMS substrates were used for cell cultures by aspirating the fibronectin solution and immediately adding cells in medium to the dish.

### Culturing and Differentiation of SH-SY5Y Cells

SH-SY5Y cells (Sigma-Aldrich), a neuroblastoma cell line, were cultured in T75 flasks in standard culture medium containing DMEM and Ham’s F12 medium in a 1:1 ratio (VWR), supplemented with 10% fetal bovine serum (FBS; lot no. 11113, Bovogen, Keilor East, VIC, Australia) and 1% penicillin/streptomycin (PS; Westburg) in an incubator at 37°C and 5% CO_2_. Cells were detached from the T75 flask using trypsin when reaching 70–80% confluency. Cells were then seeded at 1500 cells cm^-2^ at 0 DIV onto the fibronectin-coated substrates in Petri dishes in standard culture medium. All cell cultures were seeded with cells at passage 19. As the PDMS substrate did not cover the complete Petri dish, cells on the fibronectin-coated Petri dish (polystyrene) surface were used as an additional flat surface control. Cells were left to adhere to the substrates for 3 h, after which the medium was replaced with culture medium supplemented with 10 μM retinoic acid (RA; Sigma-Aldrich) for 72 h to initiate differentiation of the cells and inhibit proliferation (Dwane et al., 2013; Teppola et al., 2016). Within the 72 h, the medium supplemented with RA was refreshed after 36 h. Subsequently, at 3 DIV, the medium was replaced with culture medium supplemented with 50 ng ml^-1^ brain-derived neurotrophic factor (BDNF; Sigma-Aldrich) for 24 h to enhance neuronal differentiation (Encinas et al., 2000; Teppola et al., 2016). Cells were stored in standard culture medium until 21 DIV, with medium being refreshed every 48 h. Cultures were fixed after 21 DIV by washing the samples twice in PBS and subsequently treating them twice with 3.7% paraformaldehyde (Merck Millipore, Amsterdam, Netherlands) for 30 min.

### Immunofluorescence Staining

Immunofluorescence staining was performed on the cells covering the substrates. Anti-β-Tubulin III (Sigma-Aldrich) and anti-mouse IgG Alexa 555 (Thermo Fisher Scientific, Bleiswijk, Netherlands) were used as primary and secondary antibodies, respectively, to selectively stain the SH-SY5Y that differentiated into a neuron-like phenotype (Agholme et al., 2010). Cells were permeabilized for 10 min with 0.1% Triton X-100 (Merck Millipore). Then, they were incubation for 15 min in a blocking buffer of 10% horse serum (HS; Thermo Fisher Scientific) in PBS, followed by incubation for 1 h with 1:200 primary antibody and 1% HS in PBS and incubation for 1 h with 1:200 secondary antibody in PBS. Additionally, the cytoskeletal protein F-actin in the cells was stained using 2 drops ml^-1^ ActinGreen (Thermo Fisher Scientific) for 30 min and cell nuclei were counterstained with 2 drops ml^-^ NucBlue (Thermo Fisher Scientific) in PBS for 5 min. Samples were rinsed three times for 5 min with PBS prior to each described step.

### Image Analysis and Alignment Quantification

**FIGURE 2:**
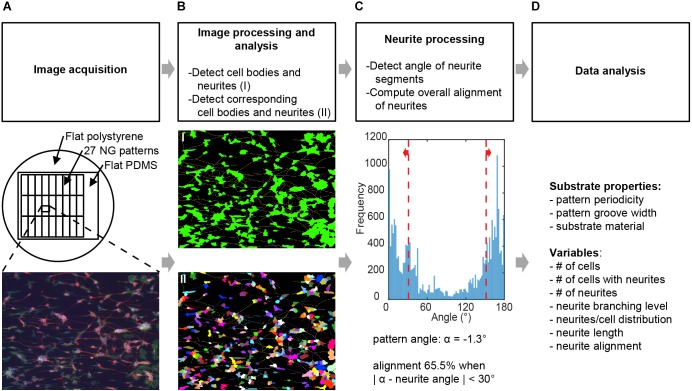
Image processing and analysis. Schematic for the screening process where **(A)** images obtained from neuronal cell cultures on different substrates are processed to deduce variables detailing neuronal culture properties. See Section “Materials and Methods” for full details. **(B)** Image processing was conducted using neuronal cell feature detection in the HCA-Vision software, which detects cell bodies and neurites and links neurites to the corresponding cell bodies. **(C)** The alignment of neurites independent of cell bodies was computed using a Frangi vesselness algorithm in MATLAB. Alignment of neurites was considered when the detected angle relative to the angle of the underlying pattern was <30°. Variables obtained from this combined analysis in **(B,C)** were number of cells, number of cells with neurites, number of neurites, neurite branching level, neurites per cell distribution, neurite length, and neurite alignment. **(D)**Variables were then analyzed for statistically significant differences between substrates and for correlation between variables.

Staining of the SH-SY5Y cells was visualized using the EVOS FL Imaging System. Images of the cell nuclei, F-actin staining, and β-Tubulin III staining were taken with a 10× objective for each pattern, the flat PDMS, and the flat polystyrene surface (Figure 2A). An additional phase contrast image with a 10× objective was taken for each experiment of each pattern name and pattern edge to obtain a reference image from which the angle of the nanogrooves could be obtained (Figure 1B).

HCA-Vision was used as a tool for quantitative analysis of images because this software specifically aims at automated analysis of neuronal cell images without requiring additional scripting (Vallotton et al., 2007; Wang et al., 2010; Figure 2B). Prior to running a batch analysis, the parameter settings for feature recognition were optimized by means of two individual images. An image was first loaded into the software, after which the user interface presented a wizard to tune a set of parameters (Supplementary Table S1) that allow for the automated and sequential detection and analysis of neuronal cell bodies, neurites, and neurite coupling to neurons. The second image was used to test the parameter set, and the entire batch of images was subsequently analyzed for each experiment. This resulted in a table with measurements taken from all the images on cell bodies and neurites. For our purposes, using this method, we determined the total number of cells, the number of cells with neurites, the number of neurites, the neurite length, and the number of neurites and branches per cell in an image.

Orientation of detected neurites for each image (Figure 2C) was calculated using a custom algorithm, which was kindly supplied by Biomedical Engineering and developed by Stefan Mariën at the Eindhoven University of Technology; this algorithm is based on the Frangi vesselness filter (Frangi et al., 1998). Algorithms based on the Frangi vesselness filter have previously been used to measure collagen fiber orientation (Foolen et al., 2012; van Spreeuwel et al., 2014), and scripts are available based on medical image improvement filters such as with 2D and 3D angiographic images (Jerman et al., 2016a,b). In brief, the Frangi vesselness filter uses the Hessian, which is a matrix of second-order partial derivatives, of a Gaussian kernel convoluted with a presented image to calculate eigenvalues and thereby locally determines vesselness likelihood. The algorithm requires parameter input in MATLAB (Supplementary Table S2) along with its Image Processing toolbox. For each image, we detected neuron bodies and neurites, and subsequently bodies and background were subtracted from the image. In this way, only the neurites are taken into account to calculate orientation. More specifically, neurite orientation is derived from the calculated vesselness likelihood of the neurites-only image for each individual pixel belonging to a neurite whose likelihood surpasses the threshold parameter (Supplementary Table S2), which allows for the calculation of the local alignment of neurites. Alignment of neurites to the underlying pattern was considered when the neurite alignment values were within the ± 30° range of the direction of the pattern, as measured from the reference images. In these reference images (Figure 1B), nanogroove direction is parallel to the edge seen underneath the pattern notation and should ideally be along the horizontal axis; however, due to manual placement of samples underneath the microscope, slight deviations occur. The direction of the nanogrooved pattern in the reference image of each pattern was obtained by calculating the angle in Fiji (Schindelin et al., 2012). For the flat PDMS and polystyrene surfaces, no actual alignment of neurites to an underlying pattern takes place. However, the assumed isotropic distribution of neurites on such flat substrates can be predetermined. We can calculate the directionality of neurites for the formed neuronal processes on flat control samples yielding an alignment value close to 33.3%. For calculation purposes, an arbitrary angle of 0° is chosen as the orientation of the underlying pattern for these flat substrates.

An alternative means of validation to the Frangi vesselness algorithm is the alignment of structures in the images, which is also calculated by FFT using the Directionality tool in Fiji (Supplementary Table S3). This tool applies Fourier spectrum analysis and computes a histogram indicating the direction for structures in an image. Additional validation of the Frangi vesselness algorithm was performed by comparison with both FFT and manual neurite measurements on a randomly selected subset of images (*n* = 3) for three substrate types: high alignment of >75%, intermediate alignment in the range of 60–75%, and flat PDMS substrates as a control. Manual measurement of the neurites was performed in Fiji on neurite-only images by drawing a straight line from the start to the end of a neurite and calculating whether the angle of this line would be within the ± 30° range of the direction of the underlying nanogrooved pattern. For the flat PDMS, the pattern angle was again assumed to be 0°.

### Statistical Analysis

Statistical analysis was performed on the data derived from the image-based screening method (Figure 2D), where cell culture experiments were conducted in duplicate and repeated for five independent cell culture batches. Data were tested for normality using Lilliefors normality test, which is a correction on the Kolmogorov–Smirnov normality test that does not require a known population mean or standard deviation. Based on the results of the normality test, data were considered to not be normally distributed. The data shown are medians with IQR unless stated otherwise. Statistically significant differences between the substrates for neurite alignment as well as neurite length were tested by the non-parametric Kruskal–Wallis test with *post hoc* Dunn’s multiple comparison test. The Kruskal–Wallis test allows for comparison between multiple independent groups that are not normally distributed. The *post hoc* Dunn’s test identifies the pairs of groups in which stochastic dominance occurs. Statistical significance was considered when *P* < 0.05. The non-parametric Spearman’s correlation test was used between output variables to test for correlation. Statistical significance was considered when *P* < 0.01.

## Results

### SH-SY5Y Cell Culture on Nanogrooves

In order to validate the use of automated image analysis in high-content screening of neuronal processes, cells from the SH-SY5Y neuroblastoma cell line were seeded and differentiated on 27 patterns of nanogrooved PDMS with varying pattern period and ridge width (Figures 1A,B). For distinction between the nanogrooved patterns, a notation was used where the letter D and the value following it refer to pattern period and its size in nm and the letter L and the value following it refer to the pattern ridge width and its size in nm. As an example, D450L180 refers to nanogrooves with a pattern period of 450 nm and a ridge width of 180 nm. Flat PDMS and flat tissue culture plate polystyrene substrates were used as controls. All experiments showed that SH-SY5Y cells proliferated and differentiated on the tested fibronectin-coated substrates, confirming the material’s biocompatibility and the use of an effective culturing protocol. Culture of SH-SY5Y cells was performed for a total of 21 days, including differentiation according to the culture and differentiation protocol. Cells were then fixed and stained for F-actin, β-Tubulin III and, cell nuclei. Epi-fluorescence images of the culture results qualitatively showed cells having neuronal morphology, wherein cells had developed neurites, that extend beyond the cell body length and that are essential to the formation of a neuronal network. Both cell bodies and neurites on nanogrooved PDMS preferred to align to the underlying pattern characterized by an oval shape of the cell body and neurite formation in the direction of the nanogrooves (Figures 1C,D). Flat substrates acting as controls showed no specific signs of orientation as cell bodies were less oval-shaped compared to patterned substrates and neurites formed in random directions (Figures 1E,F). Generally, flat polystyrene control samples showed noticeably larger cell bodies and less neurites compared to the results of all other groups of the experiment. These results confirm that SH-SY5Y cells sense the nanoscale surface features, which has also been seen in other studies (Brunetti et al., 2010; Tonazzini et al., 2014). Therefore, SH-SY5Y cells are a suitable choice for quantifying parameters related to neuronal processes via image-based screening tools. While it was previously observed that PEI coated nanogrooved PDMS substrates allow for such guidance effects in more complex primary cortical cells harvested from newborn rats (Xie et al., 2014), we have confirmed the ability to use the SH-SY5Y cell line as a neuronal model cell for the study of the influence of nanotopographical cues on the cell differentiation phenomena. The latter is linked to cellular mechanotransduction processes whereby it is also confirmed that nanogrooved, fibronectin coated PDMS acts as a functional biomimetic substrate.

### Applicability of Selected Image Processing and Analysis

To evaluate the effects on the morphological aspects of SH-SY5Y cell differentiation, 27 different nanogrooved substrates were screened against two types of flat surfaces. From the large dataset, multiple parameters had to be analyzed in an unbiased, automated manner. Whole image computational analysis methods such as the Frangi vesselness algorithm (Frangi et al., 1998) or a FFT directionality algorithm provide information with respect to orientation for all structures seen in an image. However, collecting data processed on a cell-by-cell basis allows for analysis of the neurite orientation separately from the cell body orientation as well as simultaneous determination of more distinct properties of SH-SY5Y differentiation, namely neurite length, number of differentiated cells from the total population, number of neurites per cell, and neurite branching. Therefore, we established a method to handle a large number of high-content images by using a combination of commercially available and free software to perform this type of study (Figure 2).

Our method consisted of the following steps. First, images of the different substrates (Figure 2A) were acquired by epifluorescent imaging using an EVOS microscope for each of the culture experiments. In the second step, central to the proposed method, collected images were batch processed for the detection of cell bodies and their respective neurites; this was performed using the neuronal cell feature detection of the commercially available HCA-Vision software (Figure 2B). Subsequently, the level of alignment of neurites to the underlying pattern was computed using the detected neurite segments, analyzing the direction of these segments in a Frangi vesselness algorithm and quantifying the percentage that fell within a ± 30° range of the pattern direction (Figure 2C). The pattern direction was determined from a reference image of each pattern (Figure 1B). Prior to running the batch process for the detection of the cell bodies and their neurites and the algorithm for computing the alignment level, parameters for both processes were optimized by the individual analysis of 2 images from each experiment (Supplementary Tables S1, S2). Finally, output parameters (Figure 2D) were analyzed and evaluated for statistically significant differences and correlations with respect to the effect that the nanogrooves had on SH-SY5Y differentiation and alignment values.

**FIGURE 3:**
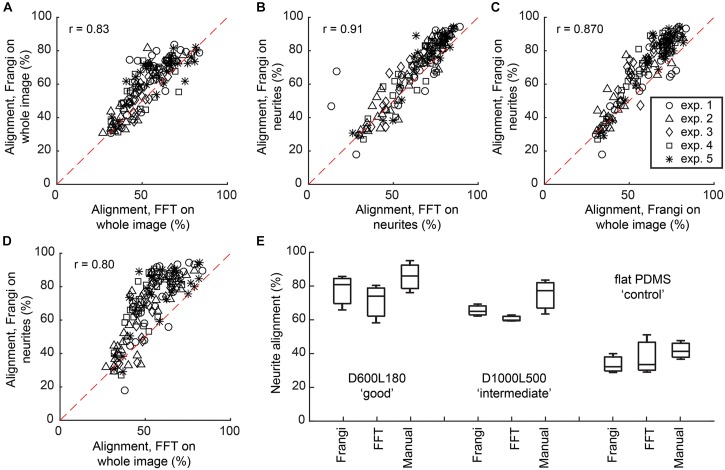
Comparison between Frangi vesselness, FFT, and manual neurite assessment. **(A)** Scatter plot showing the comparison between calculating alignment of neuronal cell culture to the underlying nanogrooved pattern by means of FFT and Frangi vesselness. Spearman’s non-parametric correlation coefficient was calculated using a two-tailed *P*-value and 99% confidence interval, resulting in *r* = 0.83 with *P* < 0.0001. **(B)** Scatter plot showing the comparison between calculating alignment of neurites to the underlying nanogrooved pattern by means of FFT and Frangi vesselness applied to a neurite-only image, resulting in *r* = 0.91 with *P* < 0.0001. **(C)** Scatter plot showing the comparison between calculating alignment of neurites to the underlying nanogrooved pattern by means of Frangi vesselness applied to a whole image versus applied to a neurite-only image, resulting in *r* = 0.87 with *P* < 0.0001. **(D)** Scatter plot showing the comparison between calculating alignment of neurites to the underlying nanogrooved pattern by means of FFT applied to a whole image versus Frangi vesselness applied to a neurite-only image, resulting in *r* = 0.80 with *P* < 0.0001. The legend in **(C)** shows markers for data of each experiment for plots **(A–D)**. **(E)** Comparison of neurite alignment as measured through Frangi vesselness, FFT, and manual neurite assessment. The comparison is made for *n* = 3 samples of pattern D600L180 for high alignment, pattern D1000L500 for intermediate alignment, and the flat PDMS substrate as a control. Box plots show median with interquartile range (IQR) and whiskers, with whiskers and median being the values found for neurite alignment for each of the different measurements on the three substrate types.

Alignment calculated via the Frangi vesselness algorithm was compared against the FFT directionality tool in Fiji (Supplementary Table S3). When comparing both methods on whole images, an overall linear trend was shown, where the calculated Spearman’s non-parametric correlation, a measure of statistical dependency between two variables described through a monotonic function, was *r* = 0.83 at *P* < 0.0001 with the alignment results being 8.2 ± 5.8% higher for the vesselness algorithm (Figure 3A). When comparing both methods on neurite-only images derived from neuronal cell feature detection, an overall linear trend was shown, where the Spearman’s non-parametric correlation was *r* = 0.91 at *P* < 0.0001 with the alignment results being 5.8 ± 5.4% higher for the vesselness algorithm (Figure 3B). Frangi vesselness for neurite-only images derived from neuronal cell feature detection were compared against Frangi vesselness for whole images, which showed an overall linear trend with a Spearman’s non-parametric correlation of *r* = 0.87 at *P* < 0.0001 with the alignment results 6.7 ± 6.4% higher for the neurite-only images (Figure 3C). Lastly, Frangi vesselness for neurite-only images derived from neuronal cell feature detection were compared against FFT for whole images, which showed an overall linear trend with a Spearman’s non-parametric correlation of *r* = 0.80 at *P* < 0.0001 with the alignment results 15.7 ± 12.0% higher for the neurite-only images (Figure 3D). These values confirm that the vesselness algorithm will give equivalent results, but they are more sensitive to alignment calculation via FFT, which was used by other authors (Johansson et al., 2006; Tonazzini et al., 2014).

Neurite alignment calculated via the Frangi vesselness algorithm was also compared against both FFT and manual measurements for a randomly selected subset of the images (*n* = 3) for three different substrates: D600L180, D1000L500, and flat PDMS (Figure 3E). For D600L180, both Frangi and FFT showed a median alignment of >75%. Manually measuring the neurite alignment resulted in a median of 85.0%. For D1000L500, Frangi showed a median of 64.6% alignment and FFT a median of 59.6% alignment. The manual measurement resulted in a median alignment of 76.6%. Lastly, for the flat PDMS control substrate median neurite alignments of 32.8% for Frangi, 34.1% for FFT, and 41.7% for manual measurement were determined.

To summarize, quantitative data analysis on parameters detailing cell and neurite properties by both a neurite-only and a whole image approach were obtained using three different methods. To demonstrate the validity of the proposed combination of neuronal cell feature detection and vesselness algorithm for describing the properties of neuronal processes on nanogrooves, results from experiments with differentiated SH-SY5Y cells were statistically analyzed as described in the following subsections.

### Nanogrooved Patterns Align Neurites

Previous studies showed that nanogrooves facilitate the alignment of neuronal outgrowths (Rajnicek et al., 1997; Johansson et al., 2006; Bremus-Koebberling et al., 2012; Xie and Luttge, 2014). A suitable analysis method must be selected for harvesting results of cellular responses. To validate our image-based screening method in such pattern optimization studies, the method should be capable of detecting subtle differences in neuronal response to pattern dimensions within the performed experiment. Additionally, the method must be able to detect specific interactions between the outgrowth and the underlying pattern.

**FIGURE 4:**
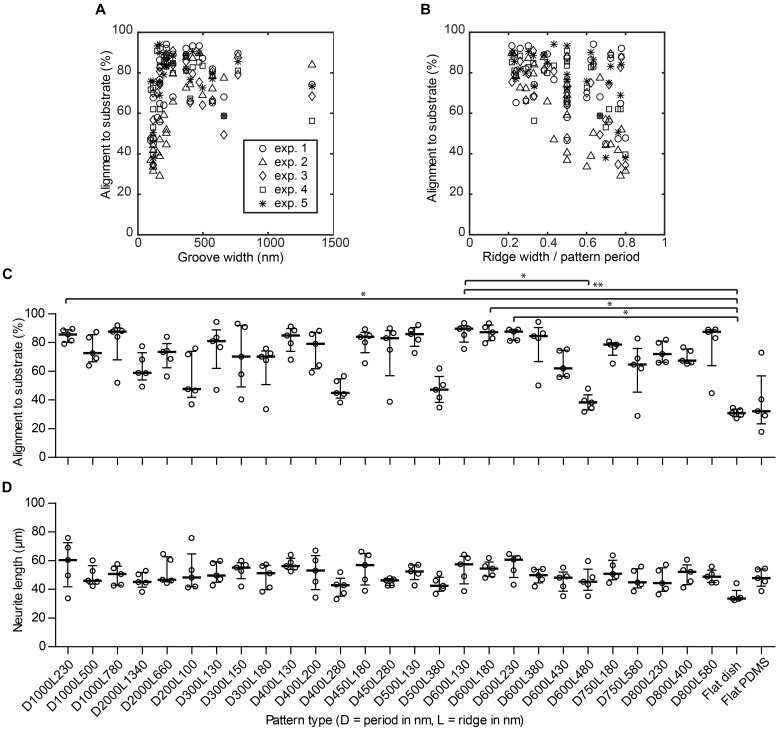
Nanogrooved patterns affect neurite alignment but not neurite length. **(A)** Scatter plot showing the percentage of alignment depending on the groove width of the nanogrooved patterns. **(B)** Scatter plot showing how alignment of neurites is affected by the ratio of pattern ridge width to pattern period size. The legend in **(A)** shows markers for data of each experiment for plots **(A–B)**. **(C)** The percentage of neurites from differentiated SH-SY5Y cells aligned to the underlying nanogrooved PDMS substrate or flat substrate (indicated underneath **D**) in an image as analyzed by neuronal cell feature detection in HCA-Vision combined with a Frangi vesselness algorithm in MATLAB. At isotropic distribution of neurites, alignment will be 33%. Data points shown are alignment percentages per substrate type from *n* = 5 experiments. Bars indicate median ± IQR. Statistically significant differences were measured using the Kruskal–Wallis non-parametric test with *post hoc* Dunn’s multiple comparison test at a significance level of 0.05. ^∗^Represents *P* < 0.05 and ^∗∗^represents *P* < 0.01. **(D)** The neurite length for neurites of differentiated SH-SY5Y cells per substrate type. Data points shown are mean neurite lengths per substrate type from *n* = 5 experiments. Bars indicate median ± IQR. Statistically significant differences were not found using the Kruskal–Wallis non-parametric test with *post hoc* Dunn’s multiple comparison test at a significance level of 0.05.

As indicated in Section 3.2, alignment of neurites to the nanogrooves is considered when neurite outgrowths have a direction that is within the ± 30° range of the nanogroove direction as measured from a reference image. By means of this cut-off definition for alignment, the calculation of neurite alignment values for flat control substrates results in some level of directionality yielding 33.3% on average. The medians for the flat PDMS and flat polystyrene substrates are 30.7% with an IQR of 27.2–34.3% and 32.2% with IQR of 17.7–73.0%, respectively (Figure 4C). These results show that indeed the flat substrates do not guide neurites. Cells in the images taken from flat PDMS show a slightly greater variation with respect to this definition of alignment values, compared to very stiff flat polystyrene. However, flat PDMS will serve as a better control against nanogrooved PDMS given that the fundamental material properties are the same for these substrates. Patterns that showed a percentage of alignment >85% are D1000L230, D1000L780, D500L130, D600L130, D600L180, D600L230, and D800L580. The majority of other patterns had alignment percentages between 50 and 85%. However, patterns D200L100, D500L380, D400L280, and D600L480 had alignment scores below 50%. Statistically significant differences at *P* < 0.05 were found for alignment on D1000l230, D600L230, and D600L180 with respect to the flat polystyrene substrate, for D600L130 with respect to D600L480 and at *P* < 0.01 for D600L130 with respect to the flat polystyrene substrate.

When looking at the distinct effect of a specific nanogrooved pattern on alignment, it can be observed that several types of patterns with periods in the range of 400–600 nm and 1000 nm show relatively high alignment values compared to other pattern periods. Hence, the patterns with a statistically significant difference for neurite alignment values compared to the flat polystyrene substrate are part of this range of patterns. However, it is remarkable that some of the nanogrooved patterns belonging to this range show the poorest alignment results, namely patterns D400L280, D500L380, D600L430, and D600L480. With regard to pattern ridge width, alignment seems to be consistently higher and with a low dispersion for ridge widths in the range of 130–230 nm compared to other values of the ridge width. On the other hand, for most patterns, neither pattern period nor ridge width seem to dominate the resulting strength of neurite alignment. These observations are put in perspective when the groove width is plotted against the alignment results (Figure 4A). Here, the width of the groove is determined by the dimensions of the pattern period minus the ridge width. Further, the ratio between ridge width and pattern period calculated as _L/D_ against the alignment results are plotted in Figure 4B. For groove widths ≤220 nm, the alignment of neurites is both lower and more dispersed. Inversely, this means for ridge widths that are relatively small compared to the pattern period, the neurite alignment is consistently high. When the ridge width becomes relatively large compared to the pattern period, resulting in a high ridge width to period ratio, neurite alignment varies greatly from either relatively strong alignment >80% to no specific alignment ≈ 33%. However, particularly for the patterns where the ratio is <0.3, the strength of alignment does not drop below 60%. These quantitative findings reinforce the suggestion made previously that ridge width to period ratio is an indicator of the capacity of a pattern to align neuronal cells (Johansson et al., 2006; Xie and Luttge, 2014).

### Effectiveness of Method on Quantifying Neuronal Differentiation

#### Neurite Length

The median neurite length (Figure 4D) for all patterns studied was within the 33.5–60.6 μm range, showing similar lengths compared to those found in the literature (Dwane et al., 2013). The longest neurites were observed for pattern D600L230. The shortest neurites were observed for the flat polystyrene substrates. No statistically significant differences were found when comparing the 27 different patterns and controls for neurite length. However, correlating the neurite lengths for each pattern with the pattern properties revealed that neurite length tends to decrease when the ratio of ridge width to pattern period increases, as Spearman’s non-parametric correlation coefficient was found to be *r* = -0.337 with *P* < 0.0001.

#### Neuronal Polarity

**FIGURE 5:**
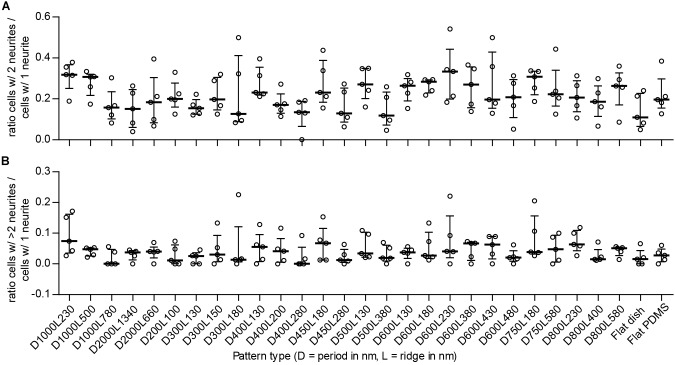
Ratio between detected types of neuronal polarity. To detect potential shifts between neuronal polarity types for the differentiated SH-SY5Y cells due to the underlying nanotopography, the ratio between cells of different polarities is displayed. Cells with 1 neurite, 2 neurites, and >2 neurites are indicative of unipolar, bipolar, and multipolar neuronal cells, respectively. **(A)** Ratio of multipolar to unipolar cells per substrate type (as indicated underneath **B**). **(B)** Ratio of bipolar to unipolar cells per substrate type. For both **(A,B)**, data points shown are ratios per substrate from *n* = 5 experiments. Bars indicate median ± IQR. Statistically significant differences were not found using the Kruskal–Wallis non-parametric test with *post hoc* Dunn’s multiple comparison test at 0.05 significance level.

The number of neurites per cell is an indicator of differentiation with regard to neuronal polarity. Neuronal polarization refers to the anisotropic distribution of neuronal cytoskeletal components to create neurites that upon further differentiation will result in axons and dendrites. Cells develop either 1, 2, or more neurites, which indicates a resemblance with unipolar, bipolar, and multipolar neuronal cells, respectively. If nanogrooves have an influence on neuronal polarity, a shift in the ratio of the polarity of cells will be recorded. The ratio of bipolar (B) to unipolar cells (U), identified by the image screening method and calculated as _B/U_, showed median values that are within the range 0.11–0.33 for all substrates (Figure 5A). Patterns with cell type ratio >0.3 were D1000L230, D1000L500, D600L230, and D750L180. The other patterns were within the range 0.15–0.3 with the exception of patterns D300L180, D400L280, D500L380, and flat polystyrene substrate, which show an even lower ratio of between 0 and 0.15. Although the median values of the ratios changed between substrates, the IQRs largely overlapped and no statistically significant differences were found. Still, it is shown that the ratio of bipolar to unipolar cells tends to decrease when the ratio of ridge width to pattern period increases, as Spearman’s non-parametric correlation coefficient was found to be *r* = -0.290 with *P* < 0.001.

The ratio of multipolar (M) to unipolar cells, calculated as _M/U_, showed median values within the range 0 to 0.074 for all substrates (Figure 5B). Patterns with this cell type ratio >0.05 were D1000L230, D400L130, D450L180, D600L380, D600L430, D800L230, and D800L580 and may demonstrate some tendency to shift in cell polarity compared to most other patterns that are within the range 0.01–0.05, with the exception of patterns D1000L780 and D400L280, which both had a median value of 0. Similar to the bipolar to unipolar cell ratio, the median values of the multipolar to unipolar cell ratio changed between substrates, but IQRs largely overlapped and no statistically significant differences were found. Calculating the correlation of the ratio of multipolar to unipolar cells and pattern properties showed that the ratio of multipolar to unipolar cells tends to decrease when the ratio of ridge width to pattern period increases, as Spearman’s non-parametric correlation coefficient was found to be *r* = -0.241 with *P* < 0.01. Overall, most differentiated cells were identified as unipolar, with bipolar and multipolar cells having far smaller populations.

#### Neurite Branching

**FIGURE 6:**
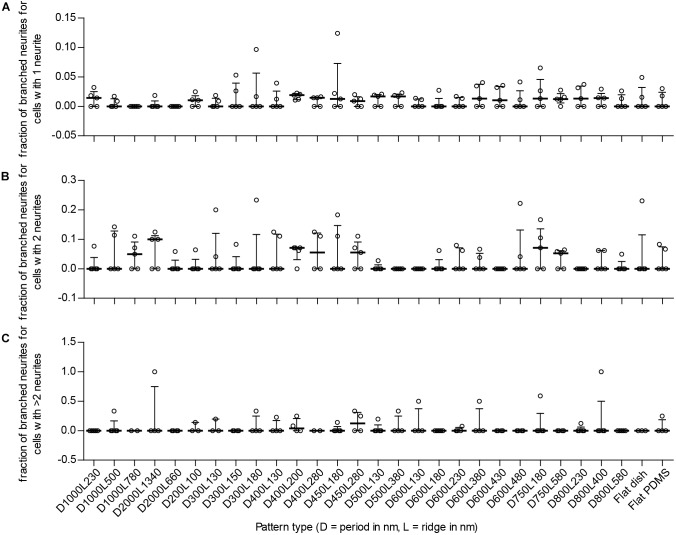
Fraction of cells with branching neurites per detected type of neuronal polarity. To detect potential enhanced differentiation of SH-SY5Y cells due to the underlying nanotopography, the fraction of branching neurites per neuronal polarity type is displayed. Cells with 1 neurite, 2 neurites, and >2 neurites are indicative of unipolar, bipolar, and multipolar neuronal cells, respectively. **(A)** Fraction of branched neurites for unipolar cells per substrate type (as indicated underneath **C**). **(B)** Fraction of branched neurites for bipolar cells per substrate type (as indicated underneath **C**). **(C)** Fraction of branched neurites for multipolar cells per substrate type. For **(A–C)**, data points shown are fractions per substrate type from *n* = 5 experiments. Bars indicate median ± IQR. Statistically significant differences were not found using the Kruskal–Wallis non-parametric test with *post hoc* Dunn’s multiple comparison test at 0.05 significance level.

Branching of neurites is another parameter that details differentiation of neuronal cells because these are important for creating connections between cells in the neuronal network. To see whether nanogrooves had an impact on branching of neurites in our experiment, the number of differentiated cells with branching neurites was compared to the total number of differentiated cells per polarity type. For 14 of the 27 patterns and controls, the median fraction of cells with branched neurites for unipolar cells was within the range 0.011–0.019 (Figure 6A). The median fraction of cells with branched neurites for bipolar cells was within the range 0.050–0.10 for 7 of the 29 substrates (Figure 6B). The median fraction of cells with branched neurites for multipolar cells was 0.042 for pattern D400L200 and 0.13 for D450L280 (Figure 6C). For all other patterns, the experiments showed no branching of neurites regardless of polarity. Overall, no statistically significant differences with respect to branching and polarity nor statistically significant correlations for branching with respect to pattern properties were found for SH-SY5Y cells between the substrates.

#### Interplay Between Neurite Alignment and Neuronal Differentiation

**FIGURE 7:**
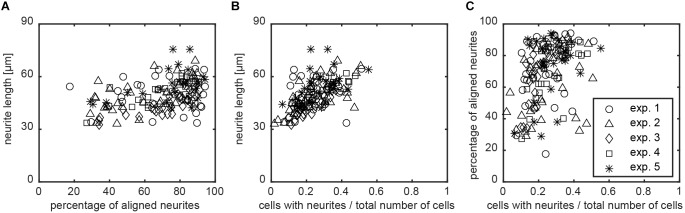
Relation between neurite alignment, neuronal differentiation, and nanogrooves. To study whether neurite alignment and differentiation of the SH-SY5Y cells on nanogrooved patterns correlate and whether the pattern structure had an influence on these properties, scatterplots were used to visualize the relation between these parameters. **(A–C)** Data for which Spearman’s non-parametric correlation coefficients were calculated using a two-tailed *P*-value and 99% confidence interval. For all cases shown, *P* < 0.01 holds true, where graphs are neurite length versus alignment with correlation coefficient *r* = 0.746 **(A)**, neurite length versus differentiated cells in total population with *r* = 0.814 **(B)**, and alignment versus differentiated cells in total population with *r* = 0.731 **(C)**. The legend shows markers for data of each experiment for plots **(A–C)**.

The image screening method computes multiple variables to detail the morphological properties of SH-SY5Y cells after differentiation into neurons. Additionally, the data analysis also provides relations between the different input and output variables. Previous subsections have demonstrated that the extent of alignment can differ between nanogrooved substrates, such as pattern D500L130 with >85% alignment and pattern D500L380 with <50% alignment. This suggests that the ratio of ridge width to pattern period is a driving factor behind the ability of a pattern to align (Johansson et al., 2006; Xie and Luttge, 2014). The ratio may also increase differentiation as specified by neurite length, polarity types, and branching level. Although the findings amongst these parameters and the strength of alignment in our study are not statistically significant using the Kruskal–Wallis test, it is still possible to find correlation for these output variables with respect to the chosen substrates and their strength of inducing alignment. The latter is also useful to consider when comparing experimental results or determining outliers. Hence, Spearman’s non-parametric correlation coefficient *r* was calculated to study the interplay between alignment and differentiation as considered by cells that formed at least one neurite. For each image, the data of alignment were first compared to the mean neurite length for that image (Figure 7A). For this correlation, *r* = 0.746 with *P* < 0.01 was found. Subsequently, for each image, the ratio of differentiated SH-SY5Y cells over all SH-SY5Y cells was also compared to the mean neurite length for that image (Figure 7B). Here, *r* = 0.814 with *P* < 0.01 was found. The correlation for the ratio of differentiated SH-SY5Y cells that formed at least one neurite over all SH-SY5Y cells versus neurite alignment was calculated (Figure 7C). For this correlation, *r* = 0.731 with *P* < 0.01 was found.

## Discussion

Our new method, which combined neuronal cell feature detection and the Frangi vesselness algorithm for the quantification of nanotopographical influence on neuronal differentiation and alignment, was presented here as an effective and sensitive way to perform automated data analysis on a large dataset. Relevant parameters, namely neurite alignment, percentage of differentiated cells, neurite length, cell polarity, and neurite branching level, were monitored efficiently, and this method ensures that the analysis is minimally biased by the experimenter or the outcome found only within a specific experimental batch. The capability to compare large datasets collected from the images in this screening method allows for quantitative gathering of high-content information with statistical relevance on the basis of differences and relations among pattern properties, alignment, and differentiation output parameters. Studies on neuronal network architecture, mechanotransduction, and neuritogenesis can benefit from such a robust, user-independent, automated method of image analysis that conveys information based on individual cells and their respective neurites.

The feasibility of the screening method was confirmed using chemically differentiated SH-SY5Y cells as a neuronal model cell on a range of nanogrooved substrates. We already expected that the differentiated SH-SY5Y cell responds to nanogrooves, showing that the cytoskeleton is affected by the anisotropic guidance cues by aligning its cell body and its neurites to the direction of the nanogrooves (Tonazzini et al., 2014). However, thanks to the combined use of neuronal cell feature detection and the vesselness algorithm, we showed here that alignment of neurites to nanogrooved patterns is not always of a high degree and can vary when pattern periods are similar but pattern ridge widths are varied, as is clearly the case in the substrates with a pattern period of 600 nm (Figure 4C). Considering the differences in alignment between pattern ridge widths for the same pattern period, two distinctive trends become visible (Figures 4A,B). Using patterns that have groove widths ≤220 nm results in unreliable alignment as alignment values show large spread from high to low alignment. The ratio of pattern ridge width to pattern period reveals that at lower values of this ratio, with limited influence of the actual pattern period, alignment will reliably increase. Consequently, the finding that a groove width lower than 220 nm provide a lesser degree of alignment suggests that the cells see the substrate surface as virtually flat. Hence, this observation also allows us to formulate the hypothesis that the receptor proteins involved in neurite mechanotransduction are too large to descend into grooves smaller than 220 nm, which may be an important cut-off value to trigger the extension of the growth cone into a different orientation instead of following the groove direction. The latter may be of importance when considering rational design choices for subsequent studies wherein specific differences in neuronal properties resulting from different nanotopographies can be used to drive the neuronal network configuration of a specific cell line.

More specifically, the introduced screening method calculates alignment values based on neurites alone, which is made feasible through the cell body and neurite detection mode given by the HCA-Vision software. It is important to note that alternative software capable of performing such neuronal cell feature detection is available. Alternatives include ImageJ and its distribution package Fiji and also CellProfiler and MATLAB. The use of these software would require additional scripting knowledge for an automated implementation to detect the features found using HCA-Vision.

Whole image analysis with directionality algorithms uses all visible features within an image to compute the directionality of features. However, this does not reveal the guidance effect of nanogrooves on the cell body and neurites separately. We show highly linear correlation between the Frangi vesselness algorithm and FFT when used in calculating alignment for either whole images or neurite-only images (Figures 3A,B). Correlation between the methods remains high for Frangi vesselness on neurites versus Frangi vesselness on whole images (Figure 3C) and Frangi vesselness on neurites-only versus FFT on whole images (Figure 3D). This shows that both methods are similar in their findings. However, higher alignment values can generally be found for the Frangi vesselness algorithm. When comparing the Frangi and FFT results with manual measurement for the selected samples that display >75% alignment, <75% alignment, and no alignment, the results for both algorithms are in agreement with the manual measurements. The manual method only takes the beginning and end point of a neurite into account. The algorithms both calculate neurite alignment across the whole path of the neurite from the cell body to its end point. Therefore, alignment tends to be slightly lower but more realistic when using the algorithms. Our observations highlight the usefulness of the Frangi vesselness algorithm, yielding representative neurite alignment measurements to the underlying nanogrooves. Additionally, algorithms are less labor intensive compared to manual measurements for large datasets.

An important consideration in the use of this method is cell seeding density, as high cell density within images affects cell detection using the software due to overlapping features, which in turn also has an impact on the detection of the cell types and neurites. The cell seeding density at the start of an experiment as well as the duration of the experiment are therefore important conditions to consider in any future designs of experiments due to the limitations they can impose in analysis.

Analysis of neurite length, neuronal polarity, and neurite branching show no statistically significant differences between the nanogrooved patterns. This rejects the hypothesis that differentiation of neuronal cells is significantly influenced by nanogrooved patterns compared to flat substrates. Nevertheless, some interesting trends are observed due to our method through the correlations that several parameters have with each other. These correlations were not observed in previously used qualitative methods. When considering either neurite length or cell polarity with respect to the ridge to period ratio, there is a correlation, but the strength of this is weak at *r* < 0.5. When looking at all samples regardless of pattern properties, the alignment, percentage of differentiated cells, and neurite length correlate. The latter suggests that, regardless of the selected nanogrooves, some level of alignment is achieved, the number of differentiated cells from the total population increases, and the nanogrooves will stimulate growth of neurites. These correlations highlight that this method of analysis is sensitive enough to reveal such information and that it is likely that follow-up experiments will reveal similar or even more distinct trends.

In summary, our results show that our proposed image-based screening method has the capability to quantify the degree of differentiation and the degree of alignment for SH-SY5Y cells on nanogrooved versus flat substrates in an automated fashion. Results confirm that the physical cues given by nanogrooves guide neurites as stated in other studies and that our method of analysis can quantify this guidance effect at sufficient sensitivity to measure differences between nanogrooved patterns. In particular, our method also shows quantitative results that reinforce previous suggestions that for nanogrooved patterns in the range of 200–2000 nm, a lower ridge to period ratio will consistently show an increase in alignment compared to patterns with higher ridge to period ratios. Although no statistically significant differences were found between the substrates with regard to neurite length, neuronal polarity, and neurite branching, our method is capable of detecting and quantifying these parameters and statistically significant correlation of neurite length, cell culture differentiation, and neurite alignment can be computed and confirm that nanogrooved patterns do influence neuronal differentiation. Hence, the proposed screening method clearly aids in the analysis and design of tissue microarchitecture *in vitro*.

## Author Contributions

RL acquired the funding. AB, SX, DM, J-PF, JdT, and RL conceptualized and designed the manuscript. AB, SX, and DM acquired the data. AB and J-PF analyzed the data. AB, J-PF, JdT, and RL interpreted the data. AB, SX, DM, J-PF, JdT, and RL prepared the manuscript. AB, SX, DM, J-PF, JdT, and RL contributed to the final approved version of the manuscript.

## Conflict of Interest Statement

The authors declare that the research was conducted in the absence of any commercial or financial relationships that could be construed as a potential conflict of interest.

## Abbreviations

BDNF: brain-derived neurotrophic factor
COC: cyclic olefin copolymer
DIV: days *in vitro*
DMEM: Dulbecco’s modified Eagle’s medium
ECM: extracellular matrix
FBS: fetal bovine serum
FFT: Fast Fourier transform
HS: horse serum
IQR: interquartile range
J-FIL: jet and flash imprint lithography
PBS: phosphate buffered saline
PEI: polyethylenimine
PDMS: polydimethylsiloxane
PS: penicillin/streptomycin
RA: retinoic acid

## References

[B1] Agholme L. Lindström T. Kgedal K. Marcusson J. Hallbeck M. (2010). An in vitro model for neuroscience: differentiation of SH-SY5Y cells into cells with morphological and biochemical characteristics of mature neurons. *J. Alzheimers Dis.* 20 1069–1082. 10.3233/JAD-2010-091363 20413890

[B2] Bremus-Koebberling E. A. Beckemper S. Koch B. Gillner A. (2012). Nano structures via laser interference patterning for guided cell growth of neuronal cells. *J. Laser Appl.* 24:042013 10.2351/1.4730804

[B3] Brunetti V. Maiorano G. Rizzello L. Sorce B. Sabella S. Cingolani R. (2010). Neurons sense nanoscale roughness with nanometer sensitivity. *Proc. Natl. Acad. Sci. U.S.A.* 107 6264–6269. 10.1073/pnas.0914456107 20308580 PMC2851967

[B4] Dwane S. Durack E. Kiely P. A. (2013). Optimising parameters for the differentiation of SH-SY5Y cells to study cell adhesion and cell migration. *BMC Res. Notes* 6:366. 10.1186/1756-0500-6-366 24025096 PMC3847106

[B5] Encinas M. Iglesias M. Liu Y. Wang H. Muhaisen A. Ceña V. (2000). Sequential treatment of SH-SY5Y cells with retinoic acid and brain-derived neurotrophic factor gives rise to fully differentiated, neurotrophic factor-dependent, human neuron-like cells. *J. Neurochem.* 75 991–1003. 10.1046/j.1471-4159.2000.0750991.x 10936180

[B6] Ferrari A. Cecchini M. Dhawan A. Micera S. Tonazzini I. Stabile R. (2011). Nanotopographic control of neuronal polarity. *Nano Lett.* 11 505–511. 10.1021/nl103349s 21241061

[B7] Foolen J. Deshpande V. S. Kanters F. M. W. Baaijens F. P. T. (2012). The influence of matrix integrity on stress-fiber remodeling in 3D. *Biomaterials* 33 7508–7518. 10.1016/j.biomaterials.2012.06.103 22818650

[B8] Frangi A. F. Niessen W. J. Vincken K. L. Viergever M. A. (1998). *Multiscale Vessel Enhancement Filtering. in International Conference on Medical Image Computing and Computer-Assisted Intervention.* Berlin: Springer, 130–137. 10.1007/BFb0056195

[B9] Hoffman-Kim D. Mitchel J. A. Bellamkonda R. V. (2010). Topography, cell response, and nerve regeneration. *Annu. Rev. Biomed. Eng.* 12 203–231. 10.1146/annurev-bioeng-070909-105351 20438370 PMC3016849

[B10] Jerman T. Pernuš F. Likar B. Špiclin Ž (2016a). Blob Enhancement and visualization for improved intracranial aneurysm detection. *IEEE Trans. Vis. Comput. Graph.* 22 1705–1717. 10.1109/TVCG.2015.2446493

[B11] Jerman T. Pernus F. Likar B. Spiclin Z. (2016b). Enhancement of vascular structures in 3D and 2D angiographic images. *IEEE Trans. Med. Imaging* 35 2107–2118. 10.1109/TMI.2016.2550102 27076353

[B12] Johansson F. Carlberg P. Danielsen N. Montelius L. Kanje M. (2006). Axonal outgrowth on nano-imprinted patterns. *Biomaterials* 27 1251–1258. 10.1016/j.biomaterials.2005.07.047 16143385

[B13] Nguyen A. T. Sathe S. R. Yim E. K. F. (2016). From nano to micro: topographical scale and its impact on cell adhesion, morphology and contact guidance. *J. Phys. Condens. Matter* 28:183001. 10.1088/0953-8984/28/18/183001 27066850

[B14] Rajnicek A. M. Britland S. McCaig C. D. (1997). Contact guidance of CNS neurites on grooved quartz: influence of groove dimensions, neuronal age and cell type. *J. Cell Sci.* 110(Pt 2), 2905–2913. 9359873 10.1242/jcs.110.23.2905

[B15] Schindelin J. Arganda-Carreras I. Frise E. Kaynig V. Longair M. Pietzsch T. (2012). Fiji: an open source platform for biological image analysis. *Nat. Methods* 9 676–682. 10.1038/nmeth.2019.Fiji 22743772 PMC3855844

[B16] Song L. Wang K. Li Y. Yang Y. (2016). Nanotopography promoted neuronal differentiation of human induced pluripotent stem cells. *Colloids Surf. B Biointerfaces* 148 49–58. 10.1016/j.colsurfb.2016.08.041 27591570

[B17] Teppola H. Sarkanen J. R. Jalonen T. O. Linne M. L. (2016). Morphological differentiation towards neuronal phenotype of SH-SY5Y neuroblastoma cells by estradiol, retinoic acid and cholesterol. *Neurochem. Res.* 41 731–747. 10.1007/s11064-015-1743-6 26518675 PMC4824837

[B18] Tessier-Lavigne M. Goodman C. S. (1996). The molecular biology of axon guidance. *Science* 274 1123–1133. 10.1126/science.274.5290.1123 8895455

[B19] Tonazzini I. Cecchini A. Elgersma Y. Cecchini M. (2014). Interaction of SH-SY5Y cells with nanogratings during neuronal differentiation: comparison with primary neurons. *Adv. Healthc. Mater.* 3 581–587. 10.1002/adhm.201300216 24115396

[B20] Vallotton P. Lagerstrom R. Sun C. Buckley M. Wang D. De Silva M. (2007). Automated analysis of neurite branching in cultured cortical neurons using HCA-Vision. *Cytom. Part A* 71 889–895. 10.1002/cyto.a.20462 17868085

[B21] van Spreeuwel A. C. C. Bax N. A. M. Bastiaens A. J. Foolen J. Loerakker S. Borochin M. (2014). The influence of matrix (an)isotropy on cardiomyocyte contraction in engineered cardiac micro tissues. *Integr. Biol.* 6 422–429. 10.1039/C3IB40219C 24549279

[B22] Wang D. Lagerstrom R. Sun C. Bishof L. Valotton P. Götte M. (2010). HCA-Vision: automated neurite outgrowth analysis. *J. Biomol. Screen.* 15 1165–1170. 10.1177/1087057110382894 20855562

[B23] Xie S. (2016). *Brain-on-a-Chip Integrated Neuronal Networks.* Ph.D. thesis, University of Twente, Enschede.

[B24] Xie S. Luttge R. (2014). Imprint lithography provides topographical nanocues to guide cell growth in primary cortical cell culture. *Microelectron. Eng.* 124 30–36. 10.1016/j.mee.2014.04.012

[B25] Xie S. Schurink B. Wolbers F. Luttge R. Hassink G. (2014). Nanoscaffold’s stiffness affects primary cortical cell network formation. *J. Vac. Sci. Technol. B Nanotechnol. Microelectron. Mater. Process. Meas. Phenom.* 32:06FD03 10.1116/1.4900420

[B26] Yim E. K. F. Pang S. W. Leong K. W. (2007). Synthetic nanostructures inducing differentiation of human mesenchymal stem cells into neuronal lineage. *Exp. Cell Res.* 313 1820–1829. 10.1016/j.yexcr.2007.02.031 17428465 PMC2038987

