# Supplementary material for: Validation and Optimization of an Image-Based Screening Method Applied to the Study of Neuronal Processes on Nanogrooves
Source: Front Cell Neurosci. 2018 Nov 6;12:415. doi: 10.3389/fncel.2018.00415 (PMC6232373; doi:10.3389/fncel.2018.00415)
Supplement: Supplementary file 1 [file Table_1.DOCX]

Supplementary Materials

Validation and optimization of an image based screening method applied to the study of neuronal processes on nanogrooves

Alex J. Bastiaens^1*^, Sijia Xie^2^, Dana A.M. Mustafa^1^, Jean-Philippe Frimat^1^, Jaap M.J. den Toonder^1^, Regina Luttge^1*^

^1^Microsystems Group and ICMS Institute for Complex Molecular Systems, Department of Mechanical Engineering, Eindhoven University of Technology, Eindhoven, the Netherlands

^2^MESA+ Institute for Nanotechnology, University of Twente, Enschede, the Netherlands

*** Correspondence:**Alex Bastiaens and Regina Luttge
[a.j.bastiaens@tue.nl](mailto:a.j.bastiaens@tue.nl) and [r.luttge@tue.nl](mailto:r.luttge@tue.nl)

Supplementary tables:

Table S1 page 1

Table S2 page 3

Table S3 page 4

| Table S1 Parameter settings for HCA-Vision^©^ image analysis of immunofluorescence images of SH-SY5Y cells on nanogrooves | |
| --- | --- |
| *Step in wizard configuration* | *Parameter value* |
| Neuron Body Detection Wizard |  |
| Select an Input Channel | 1 |
| Smoothing (Gaussian Filtering) | 6 |
| Background Correction (Morphological Top Hat) | 0 |
| Suppression of Neurites (Morphological Opening) | 6 |
| Intensity Thresholding | 0.25 |
| Nucleus Channel |  |
| Nucleus Image Available | Check box |
| Nucleus Channel | 2 |
| Nucleus Thresholding Sensitivity | 0.45 |
| Nucleus Area Threshold | 40 |
| Maximum Nucleus Size | 50 |
| Declump | Check box |
| Nucleus Selection |  |
| Intensity Threshold | 0 |
| Channel | 0 |
| Low Intensity Threshold | 0 |
| High Intensity Threshold | 255 |
| Channel | 0 |
| Object Selection |  |
| Border Width | 2 |
| Minimum Neuron Body Area | 0 |
| Optional Measurements |  |
| Additional Intensity Channel Available | Check box |
| Intensity Channel | 0 |
| Calculate Cytoplasm Intensity | Check box |
| Calculate Nucleus Intensity | Check box |
| Calculate Membrane Intensity | Check box |
| Neurite Detection Wizard |  |
| Select An Input Channel | 0 |
| Preprocessing (Gaussian Smoothing) | 4 |
| Linear Feature Detection |  |
| Linear Window Size | 17 |
| Contrast | 5 |
| Remove Small Objects | 10 |
| Gap Closing |  |
| Distance | 9 |
| Quality | 50 |
| Neurite Analysis |  |
| Debarb Small Neurite Branches | 16 |
| Thicken Neuron Bodies | 1 |
| Remove Small Trees | 4 |

| Table S2 Parameter settings for FOAtool for image analysis of SH-SY5Y neurite orientation on nanogrooves | |
| --- | --- |
| *Step in algorithm configuration* | *Parameter value* |
| Channel selection | G (for green image channel) |
| Parameters |  |
| Scales | [1,2,3] |
| Vesselness threshold | 0.9 |
| Export | Check boxes for all results |

| Table S3 Parameter settings for Directionality tool in Fiji for image analysis of SH-SY5Y cell orientation on nanogrooves | |
| --- | --- |
| *Step in algorithm configuration* | *Parameter value* |
| Method | Fourier components |
| Nbins | 180 |
| Histogram start | 0 |
| Build orientation map | Uncheck box |
| Display color wheel | Uncheck box |
| Display table | Check box |
| Debug | Uncheck box |
